# Counseling on lifestyle habits in the United States and Sweden: a report comparing primary care health professionals’ perspectives on lifestyle counseling in terms of scope, importance and competence

**DOI:** 10.1186/1471-2296-15-83

**Published:** 2014-05-03

**Authors:** Lars Weinehall, Helene Johansson, Julie Sorensen, Lars Jerdén, John May, Paul Jenkins

**Affiliations:** 1 Department of Public Health and Clinical Medicine, Faculty of Medicine, Epidemiology and Global Health, Umea University, S-901 87 Umeå, Sweden; 2 Bassett Healthcare Network Research Institute, One Atwell Road, Cooperstown, NY 13326, USA; 3 Dalarna County Council, Unit of Research and Development, Box 712, S-791 29 Falun, Sweden

**Keywords:** Attitudes, Counseling, Guidelines, Health promotion, Life style, Prevention, Primary care, Sweden, USA

## Abstract

**Background:**

The role of primary care professionals in lifestyle counseling for smoking, alcohol consumption, physical activity, and diet is receiving attention at the national level in many countries. The U. S. and Sweden are two countries currently establishing priorities in these areas. A previously existing international research collaboration provides a unique opportunity to study this issue.

**Methods:**

Data from a national survey in Sweden and a study in rural Upstate New York were compared to contrast the perspectives, attitudes, and practice of primary care professionals in the two countries. Answers to four key questions on counseling for tobacco use, alcohol consumption, physical activity, and eating habits were compared.

**Results:**

The response rates were 71% (n = 180) and 89% (n = 86) in the Sweden and the U.S. respectively. U.S. professionals rated counseling "very important" significantly more frequently than Swedish professionals for tobacco (99% versus 92%, p < .0001), physical activity (90% versus 79%, p = .04), and eating habits (86% versus 69%, p = .003). U.S. professionals also reported giving "very much" counseling more frequently for these same three endpoints than did the Swedish professionals (tobacco 81% versus 38%, p < .0001, physical activity 64% versus 31%, p < .0001, eating 59% versus 34%, p = .0001). Swedish professionals also rated their level of expertise in providing counseling significantly lower than did their U.S. counterparts for all four endpoints. A higher percentage of U.S. professionals expressed a desire to increase levels of counseling "very much", but only significantly so for eating habits (42% versus 28%, p = .037).

**Conclusions:**

The study demonstrates large differences between the extent that Swedish and American primary care professionals report being engaged in counseling on lifestyle issues, how important they perceive counseling to be, and what expertise they possess in this regard. Explanations might be found in inter-professional attitudes, the organization of healthcare, including the method of reimbursement, traditions of preventive healthcare, and cultural differences between the two countries. Further studies are needed to explore these questions, with the aim of facilitating improved lifestyle counseling in primary care.

## Background

Despite all the well-formed statements and policy decisions on the importance of prevention, and despite growing evidence about economic benefits of prevention, efforts to prevent disease still remain sideshows in health care operations throughout the western world. This was the disappointing conclusion when the Ottawa Charter was evaluated 20 years after it was adopted in 1986. To achieve sustainable progress, the evaluation expressed "a need to engage individuals and communities, and those that represent them, to change public opinion and political decisions regarding the functions of health systems to include stronger emphasis on, and greater investment in, prevention and population health interventions" [1].

Decades of experience in Swedish child and maternal preventive healthcare have convinced many that primary care should be more population-focused. Prevention was also clearly supported in the 1982 National Health Care Act. But in practice, the results were modest compared with the expectations.

Initiatives to support a re-orientation of the Swedish primary health service towards increased preventive efforts came eventually from the national political level. In 2003, the Swedish Parliament passed National targets for public health, thereby encouraging healthcare professionals to further promote health and prevent diseases [2]. As a response to this, the National Board of Health and Welfare in 2011 presented National Guidelines for disease prevention [3]. These provided guidance to support patients in establishing more healthy living habits in relation to tobacco use, hazardous use of alcohol, insufficient physical activity, and unhealthy eating habits.

In the U. S., beginning in the 1920s, general routine examinations and tests had positive impacts in clinical outpatient settings, perhaps stimulated by the financial compensation given to healthcare professionals. But, as the value of nonspecific prevention and control was questioned [4], prevention efforts became more focused with targeting screening of specific conditions [5]. Inspired by a Canadian model, the Public Health Service appointed an expert panel to review the evidence for targeted preventive interventions, and in 1989 a first Guide to Clinical Preventive Services was presented. However, the revised version in 1996 identified individual support and encouragement to improve lifestyle habits to be far more effective than diagnostic tests. The Guide to Clinical Preventive Services recommendations forms the basis for the professions’ clinical work with prevention, quality of care assessments, and health education. It is also a guide for Medicare’s reimbursement for healthcare professionals when assessing compensations for implemented prevention efforts [6]. A National Prevention Council was created in 2011 to, among other things, encourage primary care professionals to play a more active role in supporting a reorientation of primary care in a more health promoting direction [7].

A national policy aimed at expanding disease prevention efforts in the U.S. created the Prevention and Public Health Fund, which is a mandatory program funded under the Affordable Care Act [8]. This program aims "to provide for an expanded and sustained national investment in prevention and public health programs to improve health and help restrain the rate of growth in private and public health care costs." (The Patient Protection and Affordable Care Act, 2010). Prevention and Public Health Funding will be directed at prevention activities on the local, state and federal level, which seek to address obesity and tobacco use and increase the use of preventive care services.

Although prevention is high on policy-makers’ agendas in a number of Western countries, no studies have reported on comparisons between U.S. and European primary care professionals’ attitudes to preventive efforts in clinical care. Health authorities in both Sweden and the U. S. are setting new guidelines for primary care disease prevention. This creates special opportunities to study and compare how healthcare professionals think and act in relation to prevention efforts. The focus of this Swedish-American research collaboration is to examine the extent to which national prevention policies have been implemented at the local primary healthcare level in each country. The present paper aims to compare attitudes to, and self-assessed competence and performance of lifestyle counseling among primary care professionals in Sweden and in Upstate New York in the U.S.

## Methods

### Similarities and differences between the health care systems

In Sweden, coordination and delivery of health care services in a given region is the responsibility of the County Councils. The Bassett Healthcare Network (BHCN) is also responsible for the coordination and delivery of these same services within their eight-county region as it progresses towards acquisition of Accountable Care Organization status.

All professionals in both the Swedish system and the BHCN are salaried. In the BHCN, there can be moderate variability in the professional’s salary based upon patient volume above or below an expected level, whereas in Sweden this variability does not currently exist. In the BHCN, there are currently no financial incentives to either the professional or their health clinic for providing counseling on prevention. In the Swedish system, the professional does not receive financial incentives for providing this counseling, but their health center will most often receive a small incentive.

### Swedish primary care professionals study setting

In Swedish primary care, physicians, district nurses and midwives are the key professionals. Although the primary care services are performed by different professionals, they all work by assignments from 21 County Councils. Primary care is mainly financed by taxes.

### U.S. primary care professionals study setting

Bassett Healthcare Network (BHCN) serves eight counties in Upstate New York (roughly 400,000 inhabitants total). In addition to six hospitals, BHCN coordinates 26 health centers. At the time of the study, the BHCN employed a total of 97 physicians, physician’s assistants, and nurse practitioners whose main responsibility was the delivery of primary healthcare.

### The Swedish survey

In 2012, the Swedish Board of Health and Welfare examined various professional groups’ attitudes to, and experience with, dealing with disease prevention methods. The survey was conducted on a random sample at the national level by Statistics Sweden using postal questionnaires. The sampling frame was the Swedish Professional Registry [9]. The professions were stratified in eight occupational categories, with 385 people in each stratum. The investigation period was from September to October 2012. Altogether, 3,000 postal questionnaires were sent. After two reminders, 1,959 valid responses were obtained, yielding a response rate of 67 percent. The overall response rate in primary care was 71% versus 62% in somatic specialist care [10]. For the two care strata combined, the response rates were 56%, 65% and 70% among physicians, nurses and midwives respectively. Statistics Sweden did not provide response rates at the level of professions or gender within primary or specialist care.

The present study includes only data from physicians (n = 53), nurses (n = 45) and midwives (n = 82) employed in primary care. In addition to variables defining gender and profession, the study is based on the four questions from the the postal questionnaire described below.

### The U.S. survey

The U.S. survey consisted of the four questions taken directly from the National Survey of Primary Healthcare in Sweden. Each of these was asked specifically with respect to tobacco use, alcohol use, eating habits, and physical activity. The questions were: 1) In general, how important do you think it is to provide advice to patients on the following lifestyle habits? 2) To what extent are you counseling patients about the following lifestyle habits in your clinical work? 3) To what extent do you have expertise in counseling patients about the following lifestyle habits? 4) As compared with your current practice, how would you like to change the extent to which you discuss the four lifestyle habits with patients? The four questions were translated and reviewed by the bi-lingual Swedish study group members in consultation with the U.S. members.

To increase professionals participation, information on the study was featured in newsletters and emails from the BHCN Research Institute Director (a physician). These promotional activities encouraged primary care professionals to participate in the study. In addition to these activities, a medical student conducted a presentation of the study aims and objectives at a BHCN meeting attended by professionals. Participating physicians were also entered into a raffle for one of three $100 gift cards. The U.S. surveys were delivered in paper form via interoffice mail in April of 2013. Two weeks after this initial mailing all 97 professionals were sent another copy of the survey with instructions not to complete it if they had already responded. Two days after this second mailing, a research assistant contacted primary care office managers to ask them to encourage their professionals to participate. Of the 97 surveys mailed out (30 to nurse practitioners, 55 to physicians, and 12 to physician’s assistants), 86 were returned (28 from NPS (93%), 48 from physicians (87%), and 10 from PAs (83%)), yielding an overall response rate of 89%.

### Data analyses of U.S. and Swedish data sets

The U.S. data were entered into Microsoft Access and then transferred into SAS for data analyses. The Swedish data were received from the Swedish Board of Health and Welfare in Excel format. These were then converted into SAS, and merged with the U. S. data for data analyses.

**Figure 1:**
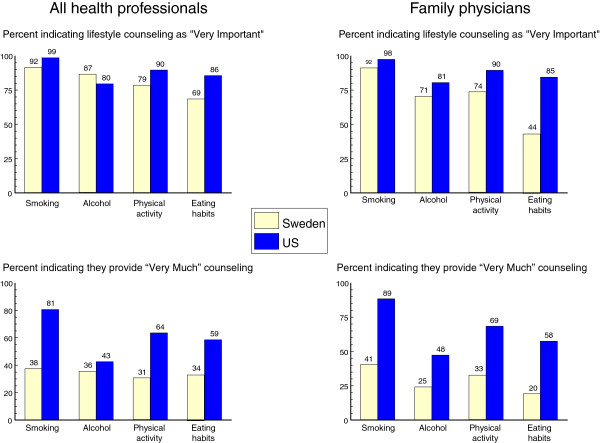
Attitudes about the importance and provision of healthy lifestyle counseling for U.S. and Swedish health care professionals.

Prior to data analyses, the response levels to the four questions were collapsed such that ‘very much’, ‘very important’ or ‘much more’ was contrasted against the aggregate of the remaining four categories. This was done under the assumption that the desired response for each question was ‘very much, ‘very important’ or ‘much more’. The proportion of professionals selecting these desired response categories was summarized versus the aggregate of the other 4 levels in tabular form for each of the four questions (Figure 1). The response levels were not collapsed into 2 categories for the question regarding the extent to which the professionals would like to change the amount of counseling he/she is giving. Levels of response outcomes were compared between the two countries using chi-square or Fisher’s Exact Test if necessary.

The difference in the professionals’ years of practice was compared between those choosing the ‘very’ category vs. some other option and between the two countries using two by two Analysis of Variance (ANOVA). These models were examined for the presence of interaction, which would be evidence of a differential relationship in the two countries.

The U.S. study was approved by the Bassett Healthcare Network Institutional Review Board. Ethical approval for the Swedish part of the study was obtained from the Regional Ethical Committee in Umeå (Dnr. 2011-64-31 M).

## Results

The results are based on 86 respondents in the U.S. and 180 in Sweden. The study results include comparisons between the Swedish and American responses for all health professionals, and also comparisons for the subset of family physicians.

### Results for the entire professionals study group

Respondents to the US survey included 35 males (4 NPs, 5 Pas, and 26 physicians) and 51 females (24 NPs, 5 PAs, and 22 physicians). Gender was missing for 1 US subject. In Sweden, the gender profile included 23 males (21 physicians and 2 nurses) and 155 females (43 physicians, 30 nurses, and 82 nurse midwives). The Swedish professionals responding to the survey had been in medical practice for significantly (p = .012) longer (20.7 years) than their U.S. counterparts (16.7 years).

Health professionals in the U.S. were significantly more likely to select the "very much" category when rating the importance of counseling for tobacco (U. S. 99%, Sweden 92%, p = .02), physical activity (U.S. 90%, Sweden 79%, p = .04), and eating habits (U. S. 86%, Sweden 69%, p = .003). The exception was seen for alcohol, with 87% of Swedish health professionals choosing "very" versus only 80% of U. S. health professionals (p = .15), Figure 1. When asked about the amount of counseling they were currently providing, the U.S. health professionals selected the "very much" level more than their Swedish counterparts. This was true for tobacco (81% versus 38%, p < .0001), alcohol (43% versus 36%, ns), physical activity (64% versus 31%, p < .0001), and eating (59% versus 34%, p = .0001), Figure 1.

**Figure 2:**
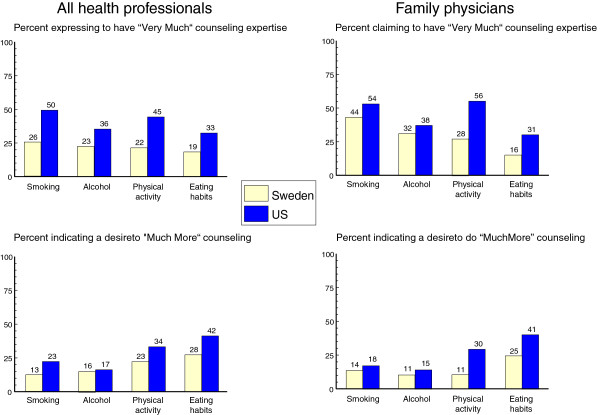
Ratings of expertise, and desire to increase counseling levels, in U.S. and Swedish health care professionals.

Swedish primary care professionals rated their level of expertise in providing primary care counseling significantly lower than their U. S. counterparts. This can be seen in the proportion of health professionals selecting the "very much" category for counseling expertise in tobacco (Sweden 26%, U. S. 50%, p = .0001), alcohol (Sweden 23%, U. S. 36, p = .02), physical activity (Sweden 22%, U. S. 45%, p < .0001) and eating habits (Sweden 19%, U. S. 33%, p = .02), Figure 2.

With regard to the desire to change the amount of counseling being given, there was a general tendency for the U.S. health professionals to express a stronger desire to increase their current levels than their Swedish counterparts. This tendency was seen for all four counseling endpoints, but was only significant for eating habits (U. S. 42%, Sweden 28% p = .037) as shown in Figure 2.

### Results for the physician subgroup

When considering only physicians, there was no significant difference in years of practice between the two countries (Sweden = 17.1 years, U. S. = 19.7 years). U.S. physicians rated counseling as "very important" more frequently than their Swedish counterparts. Although this tendency held true for all four lifestyle endpoints, it was only significant for physical activity (U. S. 90%, Sweden 74%, p = .046) and eating habits (U. S. 85%, Sweden 44%, p < .0001).

As with the results seen for all health professionals, U.S. physicians were more likely to indicate that they were providing "very much" counseling than Swedish physicians. This difference was significant for all four endpoints: tobacco (U. S. 89%, Sweden 41%, p < .0001), alcohol (U. S. 48%, Sweden 25%, p = .016), physical activity (U. S. 69%, Sweden 33%, p = .0004), and eating habits (U. S. 58%, Sweden 20%, p = .0001).

U.S. physicians more frequently reported having ‘very much’ counseling expertise than Swedish physicians for all four endpoints, but this difference was only significant for physical activity (U. S. 56%, Sweden 28%, p = .005). Concerning alcohol and smoking, the gap between the two countries in the level of expertise reported was less pronounced for physicians than for all health professionals.

With regard to the desire to change the amount of counseling, the general trend observed for the physician group was similar to that seen in the entire health professional group. The proportion of physicians wishing to increase counseling "very much" was higher for U.S. physicians for all four lifestyle categories, but only significantly so for physical activity (U. S. 30%, Sweden 11%, p = .035).

### Relationship of professionals responses to years of practice

For professional’s assessment of their expertise, those rating their expertise level as ‘very high’ had significantly greater years of experience for tobacco (P = .009), alcohol (p = .004), and physical activity (p = .006), but not for eating habits. There was no significant country by response level interactions for this factor. For the amount of counseling being given, there was no significant effect of response level for tobacco or alcohol. However, for both physical activity (p = .05) and eating habits (p = .04) there was a significant disordinal country by response level interaction effect. In Sweden for those two factors, professionals reporting giving ‘very much’ counseling had greater years in practice, whereas in the US those reporting doing ‘very much’ counseling actually had fewer years of practice compared to those choosing a different response, Table 1. With regard to the importance of counseling, no significant effects for response level or the interaction of country and response level were seen for tobacco. For the other three factors a significant disordinal interaction was seen for alcohol (p = .03), physical activity (p = .01), and eating habits (p = .02). The nature of this interaction was the same as described for the amount of counseling. Specifically, in Sweden, professionals rating counseling for these 3 factors as ‘very important’ had significantly more years in practice than professionals choosing another options. In contrast, in the US, it was the professionals with less experience rating counseling for the three factors as very important.

**Table 1:** T-test comparing years of practice between those health professionals selecting very much vs. those selecting some other option for three* of the healthy lifestyle counseling question

**Probability table**	**How important is it to provide advice?**				**To what extent are you counseling?**				**To what extent do you have expertise?**			
	***U.S.***		***Sweden***		***U.S.***		***Sweden***		***U.S.***		***Sweden***	
	**‘very’**	**‘other’**	**‘very’**	**‘other’**	**‘very’**	**‘other’**	**‘very’**	**‘other’**	**‘very’**	**‘other’**	**‘very’**	**‘other’**
*Tobacco use*	16.7	13.0	**21.4**	**12.3**	17.0	15.5	**23.2**	**19.1**	19.2	14.2	23.3	19.7
*Alcohol use*	15.6	20.9	21.1	17.4	17.6	15.9	**23.1**	**19.3**	20.0	14.9	**24.4**	**19.5**
*Physical activity*	16.0	21.8	**21.9**	**16.1**	15.5	18.6	23.1	19.6	18.3	15.3	**25.6**	**19.2**
*Eating habits*	16.3	18.8	**25.0**	**18.9**	15.6	18.2	**23.4**	**19.3**	17.1	16.4	**24.9**	**19.7**

Values in bold are significant at at least the .05 level.

*The comparison of years of practice and those selecting very much vs. other was not conducted for the question relating to changing extent of counseling, as it would be difficult to interpret what this comparison actually means without conditioning on the subjects other responses, which would create scarcity issues.

### Relationship between professionals responses for tobacco, alcohol, physical activity and eating habits

With regard to expertise in counseling, correlations between the subjects’ responses to the four lifestyle factors (tobacco, alcohol use, physical activity, eating habits) were similar between the two countries. These correlations (all significant at p < .0001) ranged from .35 to .78. Alcohol and tobacco tended to correlate the most highly (SWE .78, USA .70), while physical activity and eating habits also tended to correlate strongly (SWE .71, USA .66).

With regard to the current extent of counseling being given and the importance attached to counseling, some noteworthy differences were observed. Because of this, these differences are presented in Tables 2 and 3.

**Table 2:** Correlations between the professionals ratings of the extent to which they are currently providing counseling (All coefficients had a probability of < .05)

**SWEDEN**	**Tobacco use**	**Alcohol use**	**Physical activity**	**Eating habits**
*Tobacco use*	1			
*Alcohol use*	0.8	1		
*Physical activity*	0.67	0.61	1	
*Eating habits*	0.58	0.76	0.75	1
**U.S.**				
*Tobacco use*	1			
*Alcohol use*	0.41	1		
*Physical activity*	0.32	0.36	1	
*Eating habits*	0.27	0.48	0.76	1

**Table 3:** Correlations between the professionals ratings of the importance of counseling for each of the four lifestyle factors

	**Tobacco use**	**Alcohol use**	**Physical activity**	**Eating habits**
**SWEDEN**				
*Tobacco use*	1			
*Alcohol use*	0.59	1		
*Physical activity*	0.47	0.36	1	
*Eating habits*	0.39	0.45	0.73	1
**U.S.**				
*Tobacco use*	1			
*Alcohol use*	0.22	1		
*Physical activity*	-0.04*	0.4	1	
*Eating habits*	0.27	0.56	0.85	1

*The coefficient that is bolded and italicized does not have a probability of < .05.

For the questions regarding the extent of counseling, the correlations between the four factors were all significantly higher in Sweden (at least p < .005) except for the relationship between physical activity and eating habits, where the correlation was virtually identical (SWE .75, USA .76) in the two countries.

For importance, the correlation between the responses for the four factors was significant except for the correlation assigned to tobacco importance vs. physical activity in the U.S.. The relationship between the importance assigned to tobacco vs. both alcohol and physical activity, was significantly higher (p = .0006 and p < .0001) in Sweden (.59 and .47 respectively) than in the U.S. (.22 and -.04 respectively). Conversely, the correlation between the importance assigned to physical activity and eating habits in the U.S. (.85) was significantly higher (p = .01) than the correlation between these two factors in Sweden (.73).

## Discussion

The primary focus of this study was to provide insight into how primary care professionals in the U.S. and Sweden view the role of prevention in their clinical practice. This was reported in terms of patients’ habits with regard to tobacco use, alcohol consumption, diet and physical activity. The fact that national health authorities in both countries simultaneously have taken initiatives to enhance primary care prevention efforts represents a window of opportunity, for conducting a ‘natural experiment’.

Sweden and the U. S. have significantly different healthcare systems. However, what makes this study particularly interesting is that primary care staff at BHCN are salaried (vs. fee for service), much like their Swedish primary care counterparts. As a result, it is possible to conduct a unique international comparison that examines differences between Swedish and American primary care professionals. Understanding professionals ‘take’ on lifestyle interventions, is invaluable, as they serve on the ‘front line’ in the battle to improve health outcomes in both countries.

Based on these results, there appears to be a pronounced difference in healthy lifestyle counseling practices between the two countries, with a greater emphasis on prevention in the U.S. U.S. primary care professionals were more likely to indicate that healthy lifestyle counseling is "very important" as compared to Swedish primary care professionals. The American participants also appeared to be more vigorously engaged in healthy lifestyle counseling than their Swedish counterparts. Moreover, a greater proportion of the U.S. primary care staff claimed to have considerable expertise in lifestyle counseling and were more likely to indicate a desire to increase counseling levels. These same trends were witnessed when restricting comparisons to the physician subgroups in the two countries. Former comparisons between U.S. and European physicians are sparse, but video-recordings from consultations about hypertension from the Netherlands (1980s) and the U.S. (1990s) have been compared [11]. American doctors asked more questions and provided more information of a biomedical nature, which might be associated with more lifestyle counseling. However, U.S. consultations were longer than Dutch ones (15.4 vs 9.5 minutes), which might have influenced the content. Swedish family physicians’ consultations are by tradition at least as long as in the American tradition, which should provide time for an equal amount of lifestyle counseling.

The differences between the countries are more pronounced concerning physical activity and eating habits than for smoking and alcohol consumption. A possible explanation may be that the obesity epidemic has so far been a more tangible reality in the U.S. than in Sweden [12]. The difference between countries is less pronounced in relation to counseling for alcohol consumption. This finding may be explained by different norms between the countries concerning how personal drinking habits should be communicated in public and in healthcare settings. Another explanation might be that the Swedish Risk Drinking Project, an active national initiative during 2004 – 2010, which targeted healthcare professionals, influenced the responses of the Swedish participants [13]. However, WHO data show similar annual alcohol consumption rates in the two countries: Sweden 10.3 liters v.s. U.S. 9.4 liters pure alcohol [14]. Further, as of 2011 New York ranks below the U.S. average in consumption, falling in the 8^th^ decile (2.1 gallons annually per capita versus 2.3 nationally) [15].

One potential explanation for the differences in healthy lifestyle counseling attitudes and practices between the two countries could relate to the degree to which physicians’ are involved in national policy development. In the U.S., physicians have been integrally involved in the development of the U.S. Guide to Clinical Preventive Services for the last 30 years. This was not the case in Sweden, although physician involvement has increased in recent years. However, there continues to be uncertainty in Sweden regarding who should be responsible for population-based prevention. As recently as 1997, a governmental committee reported wide-spread agreement among physicians questioning their involvement in further preventive efforts [16].

The data which show the relationship between the amount of counseling being given for the four lifestyle factors, implies a greater degree of consistency in Sweden than in the U.S. Specifically, if a Swedish professional is providing counseling on one of the four factors, this is a much stronger indication that he is also providing counseling on the other three than is the case for a U.S. professionals. Further exploration of why Swedish health professionals tend to be more consistent in providing counseling for all four factors than their U.S. counterparts, will be addressed in a follow-up qualitative study.

Physician’s own habits have been reported to affect lifestyle counseling, as well. Specifically, physicians who do not smoke [17], who consume less alcohol and who themselves are regularly physically active are also more likely to actively engage in lifestyle counseling with their patients [18]. In contrast, the evaluation of the Swedish Risk Drinking Project indicated that doctors’ and nurses’ reported levels of alcohol consumption did not influence their discussion about harmful alcohol use among their patients [19]. This relationship could not be explored in the U.S./Swedish comparative study as information on the lifestyle habits of the respondents was not collected. For Sweden, our survey indicates that the longer a professionals has practiced, the more likely she/he would rate healthy lifestyle counseling as very important. A possible explanation for this pattern may be that many years of experience tends to underscore the need to strengthen health promotion. This result was not present in the U.S., perhaps indicating a ‘generation shift’ among American doctors. Swedish physicians were also more consistent in supporting counseling for all four lifestyle habits, while U.S. doctors tended to ‘favour’ either tobacco or physical activity and diet counseling. The recent Swedish guidelines, focusing all four habits, might have influenced the Swedish answers.

One possible explanation for the differences in these self-reported results between the two countries might be that health professionals in primary care in Sweden and the U.S. interpret the meaning of terms such as "counseling" and "advice" differently. Further, although the data from both countries are self-reported, there could be other sources of bias occurring in addition to differing perceptions. In future qualitative studies, the research team will specify the exact behaviors that health professionals are referring to when they use these terms.

This study has several noteworthy limitations, some discussed above. Further, the counseling activities were self-reported in both countries, and are therefore potentially biased. However, it seems that these outcomes are fairly in line with results from recent video-recordings of daily practice of family physicians and practice nurses in the Netherlands [20,21].

While the Swedish survey was based on a national sample, the U.S. sample population was relatively small and confined to a eight county healthcare region in Upstate NY. As a result, it cannot be said that the views of BHCN professionals necessarily represent views of the general population of all U.S. primary care professionals. BHCN’s professionals are salaried, whereas a large proportion of professionals elsewhere in the U.S. are employed in a fee for service model. How the professionals responses might vary between these two compensation modalities, if at all, cannot be predicted. Further, BHCN’s professionals serve an almost exclusively rural population, which suggests that generalizability to urban areas is not warranted. While we believe that the results generalize to salaried U.S. professionals practicing in rural areas, results of the study should primarily be viewed as hypothesis generating.

## Conclusions

The study demonstrates significant differences between the extent to which Swedish and American healthcare professionals are engaged in lifestyle counseling, how important they perceive counseling to be, and what expertise they possess in this regard. The differences between the countries may seem surprisingly large. Because the pattern is reflected among all participants, as well as among doctors, there are good reasons to try to clarify the differences in healthy lifestyle counseling attitudes and practices in the two countries. To further explore this issue, the research team will conduct qualitative studies in each country in order to get a clearer picture of what healthy lifestyle counseling means and how it is practiced. Interviews with patients in both countries will also seek to understand the patients’ comprehension of, and desire for, healthy lifestyle counseling.

## Abbreviations

U.S: United States; BHCN: Bassett Healthcare Network.

## Competing interests

The authors of this paper have no competing interests, either financial or otherwise related to the data or conclusions of this article.

## Authors’ contributions

All authors contributed to the study design and the development and testing of the survey instrument. Lars Weinehall wrote the introduction. He also assisted with the data analyses and in writing the methods, analysis, results, and discussion sections. Helene Johansson assisted in writing the introduction and discussion sections. Julie Sorensen contributed to writing the introduction and conclusions. She also assisted in the writing of the methods section. Lars Jerdén assisted in writing the introduction and assisted in writing the discussion and conclusions parts. John May assisted with writing the introduction and the conclusion sections. Paul Jenkins performed the data analyses and wrote the method and analysis sections. All authors read and approved the final manuscript.

## Authors’ information

*Lars Weinehall,* MD, PhD, is Professor in Epidemiology and Family Medicin at Umeå University.

*Helene Johansson,* PhD, is Lecturer and Research Scientist at Umeå University.

*Julie Sorensen,* PhD, is Research Scientist at Bassett Healthcare Network Research Institute.

*Lars Jerdén,* MD, PhD, is a Primary Care Physician and Research Scientist at the R&D Unit, Dalarna County Council.

*John May,* MD, is Director of the Bassett Healthcare Network Research Institute.

*Paul Jenkins*, PhD, is Research Scientist at Bassett Healthcare Network Research Institute.

## Pre-publication history

The pre-publication history for this paper can be accessed here:

http://www.biomedcentral.com/1471-2296/15/83/prepub
